# Preclinical Modeling and Therapeutic Avenues for Cancer Metastasis to the Central Nervous System

**DOI:** 10.3389/fonc.2017.00220

**Published:** 2017-09-19

**Authors:** Mohini Singh, David Bakhshinyan, Chitra Venugopal, Sheila K. Singh

**Affiliations:** ^1^McMaster Stem Cell and Cancer Research Institute, McMaster University, Hamilton, ON, Canada; ^2^Faculty of Health Sciences, Department of Biochemistry and Biomedical Sciences, McMaster University, Hamilton, ON, Canada; ^3^Faculty of Health Sciences, Department of Surgery, McMaster University, Hamilton, ON, Canada

**Keywords:** leptomeningeal metastasis, brain metastasis, *in vivo* models, metastasis, brain metastasis therapies

## Abstract

Metastasis is the dissemination of cells from the primary tumor to other locations within the body, and continues to be the predominant cause of death among cancer patients. Metastatic progression within the adult central nervous system is 10 times more frequent than primary brain tumors. Metastases affecting the brain parenchyma and leptomeninges are associated with grave prognosis, and even after successful control of the primary tumor the median survival is a dismal 2–3 months with treatment options typically limited to palliative care. Current treatment options for brain metastases (BM) and disseminated brain tumors are scarce, and the improvement of novel targeted therapies requires a broader understanding of the biological complexity that characterizes metastatic progression. In this review, we provide insight into patterns of BM progression and leptomeningeal spread, outlining the development of clinically relevant *in vivo* models and their contribution to the discovery of innovative cancer therapies. *In vivo* models paired with manipulation of *in vitro* methods have expanded the tools available for investigators to develop agents that can be used to prevent or treat metastatic disease. The knowledge gained from the use of such models can ultimately lead to the prevention of metastatic dissemination and can extend patient survival by transforming a uniformly fatal systemic disease into a locally controlled and eminently more treatable one.

## Introduction

A metastatic tumor is a secondary tumor formed from cells that have escaped from a primary tumor elsewhere in the body. Metastases are the most frequent neoplasm to affect the adult central nervous system (CNS), occurring 10 times more than primary brain tumors (1). Metastases commonly arise within the brain parenchyma, where tumor cells travel *via* the arterial circulation and are deposited at terminal “watershed areas” (2). Metastatic spread to the leptomeninges is a rare presentation of CNS metastasis, developing from the infiltration of metastatic cells into cerebrospinal fluid (CSF) encasing the brain and spine. Approximately 40% of cancer patients will develop brain metastases (BM) (3), and 5–8% will develop leptomeningeal metastases (LM) throughout the progression of the disease (4). The primary cancers that have the highest propensity to develop BM are lung (40–60%), breast (15–30%), and melanoma (5–15%) (5). Up to 24% of hematological malignancies result in LM, 10–32% from primary CNS tumors, and of solid tumors the most common origins are lung (14–29%), breast (11–64%), melanoma (6–18%), and the gastrointestinal tract (4–14%) (6–10). Both BM and LM are associated with poor clinical outcome. The median survival rate of untreated BM and LM patients is 4–6 weeks, yet even when receiving standard interventions (palliative radiotherapy and intrathecal chemotherapy) survival is merely increased to 8–16 weeks (6, 11). The incidence of BM and LM has increased in recent years due to both the improved efficacy of primary tumor interventions, which in turn increases survival and time for metastatic development, as well as the lack of available treatments that are capable of penetrating the blood–brain barrier (BBB) to target the metastatic cells (7, 12). Moreover, administration of current genotoxic treatments can select for increasingly chemoresistant metastatic populations, contributing to their growing resistance over time (7, 12, 13).

## The Metastatic Process

### Migration

A tumor cell can obtain a metastatic phenotype through several mechanisms. Briefly, this process requires a cell to undergo a (1) loss of cell–cell adhesion, (2) acquisition of motility, and (3) ability to digest through surrounding tissues to enter/exit the circulation. Different strategies can be employed by tumor cells to achieve invasive phenotypes. When intercellular junctions have been lost, cells can migrate as single entities (14). These single cells can adopt two main morphological types to promote their motility, amoeboid, and mesenchymal. Amoeboid cells are rounded or abnormally shaped and produce “bleb”-like protrusions to aid migration (13). A typically accepted mechanism to achieve a mesenchymal phenotype is through the epithelial–mesenchymal transition (EMT) (13). Initiated by external and internal factors (environmental cues, transcription factors, etc.), this transition induces a shift from an epithelial state to a more motile mesenchymal phenotype, characterized by an elongated, spindle-like form (15). Conversely, recent studies have addressed the necessity of EMT in metastasis where, due to the transient, non-linear of the process, tumor cells may not require full completion of EMT to become metastatic. Studies have shown that forced induction of EMT through overexpression of EMT-regulating transcription factors causes a loss of tumor-initiating properties in the mesenchymal tumor cell (16–18). As such, EMT has been proposed to include a spectrum of phenotypes, where a tumor cell will undergo partial phase shifts to promote migration as well as maintain their tumor-initiation capacity (19). Cells can migrate collectively with intact cell–cell contacts, where either a single cell or multiple cells will serve as a leader to a line or sheet of follower cells. Collectively migrating cells can be of either epithelial or mesenchymal phenotypes, which may differ between the leader and follower cells (15). In some cases, EMT may not be required at all to achieve migration. Utilizing a lineage-tracing Cre system, Fischer et al. determined that the majority of cells forming lung metastases were of an epithelial phenotype (20). Similar results were found by Zheng et al., using a genetically engineered model of pancreatic cancer and EMT inhibited by *SNAIL1* or *TWIST* deletion (21).

### Invasion into the Circulation

These metastasizing cells can either secrete various matrix metalloproteinases and enzymes to remodel surrounding tissue or, in the case of amoeboid cells activate contractile actin:myosin core networks to squeeze between intercellular spaces, allowing them to intravasate into the surrounding tissue, and invade adjacent blood or lymphatic vessels (22). Once in the circulation, the majority of metastasizing cells will succumb to a myriad of lethal barriers, ranging from host’s immune response to shearing forces within the vessel (23). These metastasizing cells, otherwise known as circulating tumor cells (CTCs), have adopted successful defensive strategies. One of the methods employed by single CTCs involves platelet aggregation, where the CTC will express thrombin to collect platelets and form protective layer from immune surveillance and hemodynamic shearing forces (24). Metastasizing cells that have invaded the circulation as collective groups can form clusters of CTCs or microemboli, arising from oligoclonal tumor cells and are rarer but appear to have a higher metastatic potential than single CTCs (25). These clusters provide protection similar to platelet shields but also an added benefit of avoiding anchorage-dependent apoptosis (26).

### Colonization

As the cell arrests at the new site, both through homing mechanisms as well as physical restraints (27), the cell will extravasate into the tissue. Depending on the environmental cues the cell will either remain in a dormant state or colonize the tissue where initial seeding of the brain will form micrometastases and subsequent development of tumor-associated vasculature (neoangiogenesis) will give rise to macrometastases (28). The clonality of the resulting metastasis is dependant on the nature of the seeding cell. A single cell can give rise to a clonal metastasis, whereas a polyclonal metastasis can develop from a CTC cluster or seeding of the same region by multiple single cells (29). Recent studies have identified metastases to be primarily polyclonal, including prostate (30), breast (31), and pancreatic (32), which is consistent with the concept of enhanced survival and metastatic seeding potential by CTC clusters over single metastatic cells (29).

To enter the CNS, metastasizing cells must overcome additional barriers during extravasation and colonization: the BBB and brain–CSF barrier (BCSFB). It is thought that the properties required to exit the circulation are rate limiting; though millions of cells can be shed into the circulation, only a very small percentage are able to colonize the secondary environment (33). However, once these obstacles are overcome, the CNS becomes a sanctuary site for these metastasizing cells, allowing their escape and protection from typical cytotoxic agents and immune surveillance that are unable to cross an intact BBB and BCSFB (7).

The flow of arterial blood largely determines the spread of metastatic cells throughout the brain parenchyma: 85% of BM arise within the cerebrum, 5–10% within the cerebellum, and 3–5% within the brainstem (2). The type of primary tumor can also dictate the distribution and number of metastases within the brain. For instance, lung cancers typically result in multiple lesions within the occipital lobe and cerebellum, whereas breast cancer results in metastases within the brain parenchyma, leptomeninges, cerebellum, and brain stem (5, 34). Within the meninges, LM are generated either by diffuse, non-adherent single cells or clusters or nodules (35).

**Figure 1:**
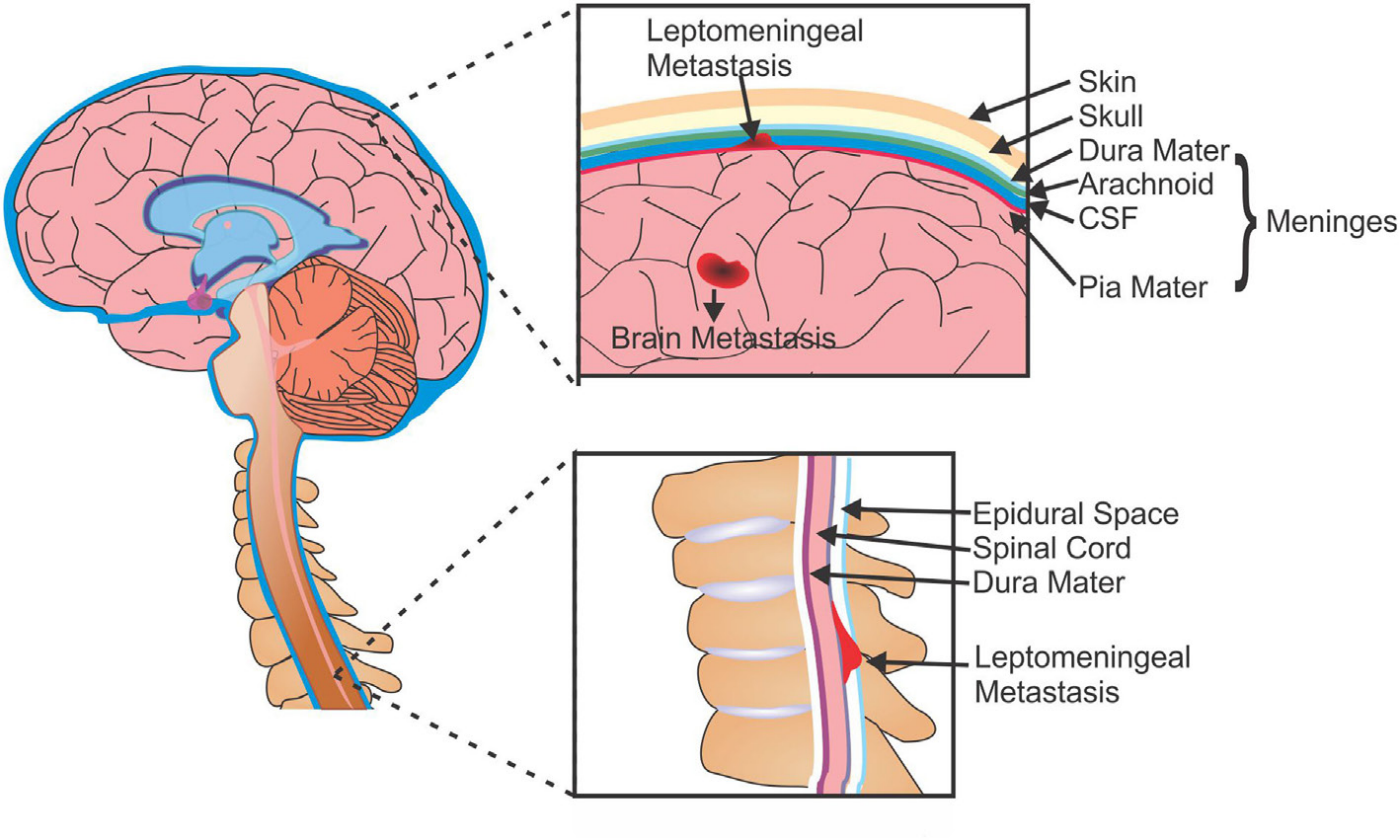
Metastases of the brain and leptomeninges. Cells from primary tumors are able to metastasize to the brain and can form secondary tumors within the parenchyma (BM), or can enter the CSF and spread to the membranes surrounding the brain and spine (LM). CSF, cerebrospinal fluid; BM, brain metastasis; LM, leptomeningeal metastasis.

Brain metastases typically develop from solid and hematological tumors, whereas LM can arise from primary CNS tumors (e.g., medulloblastoma, glioma, and PNET), systemic cancers (lymphoma and leukemia), or solid tumors. In LM, metastatic cells gain access to the CSF (and subsequently the leptomeninges) through several methods: the most common route is hematogenous or lymphatic systems; however, cells are also able to enter the CSF by escaping from adjacent bone tumors (i.e., the skull or spine) into the dural sinus or epidural plexus (36). Once in the CSF these cells can travel throughout the CNS, either remaining within the leptomeninges or invading the brain parenchyma, spinal cord, or nerve roots (Figure 1).

A significant controversy in cancer research surrounds the origin of metastatic spread; do metastases develop in a linear fashion after primary tumor formation, or in parallel to the primary tumor? The linear model of dissemination is intimated in cancers where there are close genetic similarities between the primary and secondary tumors, whereas the parallel model is suggested in cases of genetic diversity. A third theory suggests metastasis-to-metastasis seeding (37). Although from several studies the general thought appears to lean toward parallel progression of metastatic dissemination, recent phylogenetic studies have shown multiple modes of dissemination (38–42).

## Considerations for the Development of BM and LM Models

The complex nature of the metastatic process has led to the generation of several 2- and 3-dimensional *in vitro* assays that strive to recapitulate the various stages of metastasis under more stable conditions. For instance angiogenesis or neovascularization, the process of forming new blood vessels branching from existing vasculature, can be recognized through a tube formation assay, where cells plated on an extracellular matrix layer that mimics the *in vivo* environment and will form tubule-like structures that resemble vessels (43). Cancer cell migration, an integral component of metastatic cells, can be modeled in multiple assays including transwell or Boyden chambers, scratch wound, zone exclusion, or microfluidics. These assays have been invaluable as tools to not only delineate the intricacies of the mechanistic regulation of metastasis but also serve as screening platforms for therapeutic targets, unfortunately they also face several limitations. The involvement of complex host/cell interactions throughout BM development are more accurately examined *in vivo*, such as anatomical barriers (BBB and BCSFB), stromal/environmental determinants, immune signaling and response, and cytokines/growth factors (44). As such, animal models represent a vital tool in a scientist’s repertoire for translational research. A clinically relevant *in vivo* model can enable researchers to identify the genetic events that contribute to metastatic development within the CNS and provide a platform to identify and screen novel therapeutics (44).

Although the genetic mouse models have become an important tool in studying the functional significance of defined mutations in the development of BM and LM, such models lack the ability to recapitulate the genetic heterogeneity of primary human tumors. Furthermore, the genetically engineered mouse models (GEMMs) are limited by complex breeding schemes, incomplete tumor penetrance, and variable tumor onset (45). In contrast, patient-derived xenograft (PDX) models for many cancer subtypes (46–51) have been generated through injection of patient tumor cells into an appropriate microenvironment. Tumors generated through PDX models have been shown to retain the molecular identity and recapitulate the complex heterogeneity of the original patient tumor. In addition, PDX models allow for a more accurate evaluation of tumor growth patterns, metastatic properties, and their changes in response to therapeutic intervention (52, 53). Currently, various xenograft models have been developed that are capable of reproducing specific individual stages of metastasis, providing a more detailed understanding of the intricacies involved in the process. For instance, the avian embryo provides a unique model support system for many metastatic features, including growth, invasion, and angiogenesis. The chorioallantoic membrane, a vascularized embryonic tissue, shows easy engraftment of human cells, and the embryo itself provides an immunodeficient environment (54–56). The use of zebrafish xenograft models has also risen over the last few years, providing a novel high throughput and inexpensive platform for drug discovery and *in vivo* imaging (57, 58). Despite the novelty of these unique models, the use of mice and rats (murine) has remained a standby host species in modeling metastasis, providing highly reproducible disease development and easy to manipulate/inject due to size (59). The advent of transgenic and immunodeficient strains significantly increased the success rate of tumor transplantation and human–mouse xenograft model development (60).

When developing an appropriate *in vivo* model for LM and BM, several biological and technical factors must be considered.

**Figure 2:**
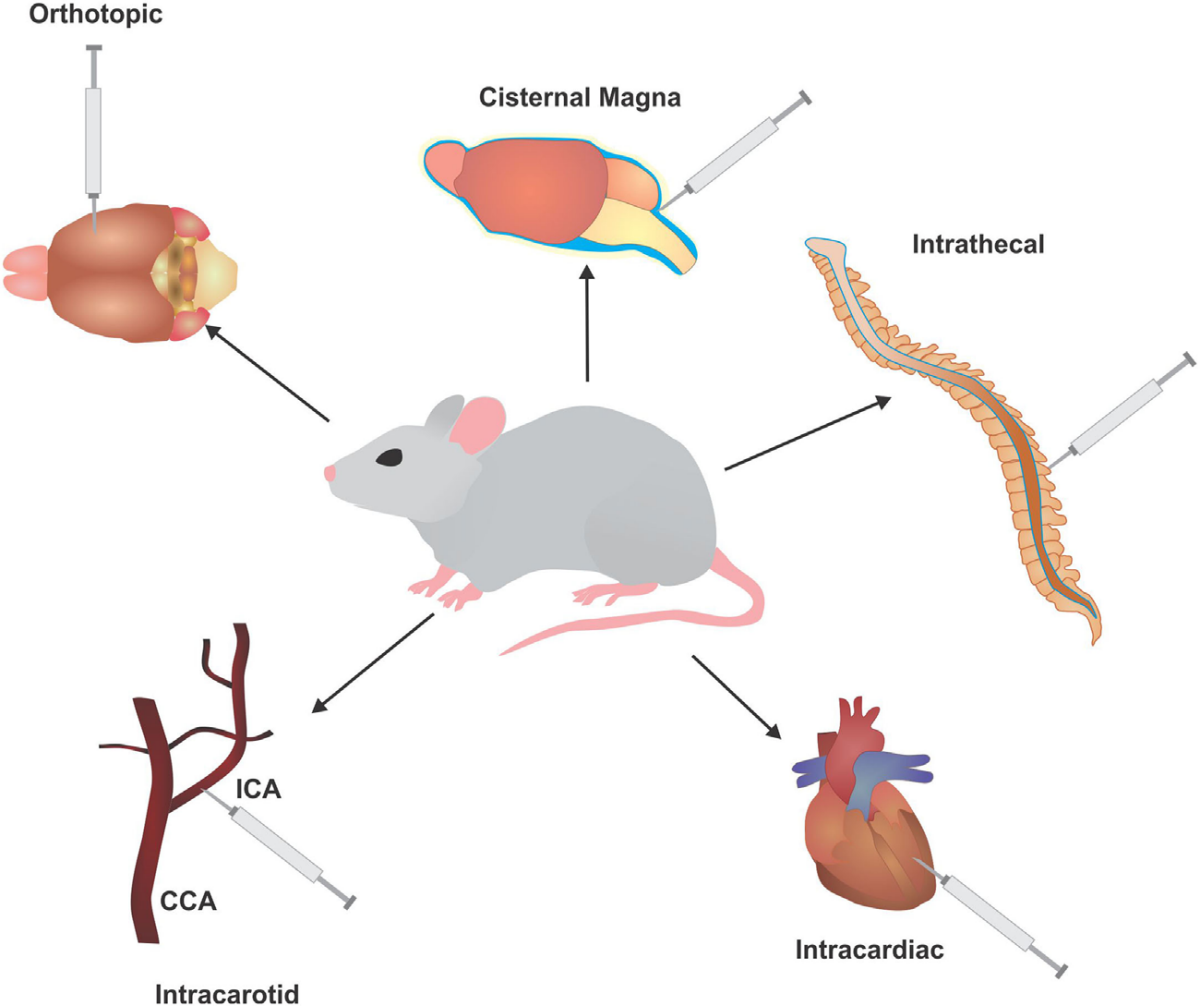
Injection routes utilized to develop *in vivo* models of leptomeningeal metastases (LM) and brain metastases (BM). Common injection routes used in murine models to develop BM and LM, typically involving injection of cells of directly into the circulation or cerebrospinal fluid to bypass the initial stages of metastasis. ICA, intracarotid artery; CCA, common carotid artery.

1. Material injected: commercial cells are a commonly utilized cell source, providing an unlimited number of cells for cost-effective use. Unfortunately, these lines have undergone significant selective pressures from years of culturing that they rarely represent the genotypic or phenotypic profile of the original patient sample and are sometimes even misidentified or cross-contaminated (61). Conversely, patient-derived cell lines provide a much more accurate representation of clonal heterogeneity existing within the original tumor, as the length of culture remains minimal, though these lines also face difficulties due to poor growth and engraftment, and a limited life span (61). Moreover, a significant concern with patient-derived cell lines is that the majority of patients have received some form of therapy, thus there are very few samples that have not faced a selection pressure from exposure to a chemotherapeutic (62).
2. The number of cells delivered, a property easily controlled by the researcher, can also play a large role in the time it takes for engraftment. A larger the cell number injected may permit for a shorter incubation period; however, this may not accurately represent the slower growth observed with the clinical presentation of metastatic progression. Conversely, a low cell number may not be engrafted easily, reducing the success rate of engraftment or cell collection (62).
3. Host selection: the choice of host when establishing a metastasis model can be key to successful engraftment rates. Murine models can be divided into two broad categories: (1) syngeneic and (2) xenogeneic. Syngeneic models utilize cancer cell lines of the same genetic background as the host and are typically generated through chemical or spontaneous induction. These models offer researchers the ability to study oncogenesis and metastatic progression in the presence of a functioning immune response and potential to identify therapeutics that can target the immune system. Unfortunately, this model is solely mouse related, which can have difficulties with correlations to human disease. On the contrary, xenograft models are developed from the administration of human cancer cells into an immunocompromised host. The lack of an immune response, which would otherwise attack the foreign cells injected and limit engraftment, permits a high rate of human tumor transplantation and study of human cancer cell behavior in a live host but lacks information on the interaction between the immune system and tumor cells.
4. The route of injection (Figure 2): the location of cell delivery and subsequent tumor engraftment and metastatic progression is another decision vital to model development. Due to circulation patterns, some locations for metastatic spread are more likely to over others, such as tail vein injections resulting primary in lung metastases (62). Certain hosts do not possess the proper/compatible physiology to represent clinical disease progression, whereas injections in some areas may not even be feasible for a particular host due to anatomical differences. Another criteria is host size, where a larger animal may allow for easy and safe repeated access to the injection route (59).

When modeling metastasis, the best route of injection would replicate tumor formation at the primary site first and subsequent metastatic development. Several such models have been established with commercial mouse and human cell lines, unfortunately this method can be laden with difficulties in capturing the metastatic cells at the desired secondary site. To overcome this, successive rounds of *in vivo* selection are performed with cells harvested from the secondary site and reinjected, selecting for cells that are aggressively metastatic with each round (63, 64).

(a) Intracardiac/intracarotid: a common method for BM development is direct injection of tumor cells into the circulation. This method is more of an assessment of brain colonization and not full metastasis, as it selectively ignores the ability of cells to undergo EMT and intravasate into the circulation. In addition, the number of cells injected into the circulation is several folds higher than the number of cells that would typically escape from the primary tumor. Nonetheless, this method allows for selection of highly metastatic populations that are able to cross the BBB and BCSF to engraft into the brain. Intracardiac injections (*via* the left ventricle) allow cells to freely enter the circulation and have indiscriminate access to all organs of the body, allowing cells to seed metastases in different areas (65). Injection of cells into the intracarotid artery allows cells to travel directly to the brain, and primarily produces BM and LM (66–69).
(b) Orthotopic: orthotopic injections place cells directly into the originating environment of the primary tumor. For BM development, the most common route of inoculation of tumor cells derived from a BM or primary brain tumor is directly into the brain parenchyma (intracranial). This surpasses all barriers encountered in the initial and mid stages of metastasis, allowing the cells to begin colonization, but creating a significant selection bias by giving cells that may not be capable of surviving the metastatic cascade an opportunity to engraft. When utilizing tumor cells from other primary cancers, the injection site will follow accordingly for better representation of the metastatic cascade. For instance, melanoma cells can be injected subcutaneously, lung cancer cells injected intrathoracically, and breast cancer cells injected into the fatpad (70–72).
(c) Intrathecal: a common method for LM modeling is intrathecal, allowing direct entry of cells into the CSF *via* the cisterna magna or spinal canal (73, 74). The larger size of a rat provides safe, easy, and repeated access to the CSF through these methods. For instance, an arachnoid catheter can be implanted into the great cistern and passed along the spinal cord (75).

Keeping these factors in mind, several groups have proven successful in developing clinically relevant models of LM and BM. Massague et al. performed several rounds of selection on human and mouse cancer lines through injection into the cisterna magna, allowing the cells to propagate within the leptomeningeal space before collecting the cells from the basilar meninges and thecal sac. After the third round of selection, cells were then injected intracardially, where hematogenously disseminated cells were found to consistently form LM as opposed to BM, faithfully replicating many clinical and histopathological aspects of LM (76). A more recent study preformed by this group utilized LM models to dissect the molecular processed involved in leptomeningeal dissemination of breast and lung cancers. They determined that cancer cells within the CSF express complement component 3 to promote disruption of the BCSF and is predictive of leptomeningeal relapse (77).

Sandén et al. were able to propagate a cell line from a primary patient sample of Group 3 MB with overexpression of c-Myc, a gene that the worst clinical outcome for MB. Intracerebellar injection of this cell line resulted in primary brain tumors that recapitulated both epigenetic and phenotypic characteristics of the original patient sample. In addition, similar to the clinical progression of the disease, researchers were able to observe metastatic spread to the meninges and down the spinal axis even before it was observed in the surviving patient, thus providing an opportunity to study early stages of spinal dissemination and allow for preclinical evaluation of targeted therapeutic interventions (45).

Recently, Singh et al. successfully established cell lines from primary patient samples of lung BMs, where specific *in vitro* culture conditions were utilized to enrich for a metastatic subpopulation of cancer stem cells within these BM, termed brain metastasis-initiating cells (BMICs). Using these BMICs, they developed a novel PDX model of lung-to-brain metastasis, where through intracardiac injections the researchers were able to obtain macrometastases, whereas intrathoracic injection of BMICs not only reformed tumors within the lung but also developed micrometastatic growths within the brain (78).

## Modeling Therapy Response

Originally described in 1800s (79, 80), few substantial advancements have been made since then in understanding the progression of LM, and this progress has added very little to the improvement upon the dismal survival rates for patients. A similar lack of therapeutic progress is seen with BMs. The typical treatment strategy for BM and LM is palliative care for poor risk patients, as treatment options offering any significant extension of survival are presently not available. Poor risk patients may receive radiation therapy, analgesics and corticosteroids for persistent pain and headaches, antidepressants, antinauseants, and anticonvulsants (36). Good risk patients will receive treatments tailored to control the tumor, including stereotactic radiosurgery, whole-brain radiotherapy, surgical resection, and systemic treatments (81). Systemic therapies include chemotherapies, small molecules (Table 1), and immunotherapies and can be administered depending on various patient factors, such as tumor histology and the patient’s prior treatment history to theoretically target both the active systemic disease as well as the LM and BM (7). Intrathecal administration (direct injection into the spinal canal) of anticancer agents guarantees the treatment will enter the CSF; however, this route can have limited efficacy. An alternative intraventricular administration shows improved CSF drug levels, especially in bulky tumors, and less variability between patients (7).

1. Chemotherapy: various chemotherapeutic agents have been employed to treat LM and BM, often used in a combination of 2–3 along with whole-brain radiotherapy. For BM, standard chemotherapies are administered based on the primary cancer. For instance cisplatin, cyclophosphamide, etopside, prednisone, and irinotecan have all been administered for BM and LM of lung, breast, and melanoma cancers (81). Capecitabine, belonging to the class of fluoropyrimidines of chemotherapies, is administered for several cancer types (106), and a phase II trial has shown the combination of lapatinib and capecitabine as a first line treatment for HER2-positive breast cancer (89). Temozolomide has shown a modest therapeutic effect when administered alone, however shows much more promise when used in conjunction with whole-brain radiotherapy and/or other anticancer agents, as discussed in a thorough review by Zhu et al. (107). For LM, standard chemotherapies administered are methotrexate, cytarabine (Ara-C), thiotepa, all safe for intrathecal administration (108).
2. Small molecules: the mutational status of the primary tumor can determine the type of small molecule administered to BM and LM. Tyrosine kinase inhibitors (TKI) such as gefitinib, osimertinib, and erlotinib target EGFR mutations found in lung cancers, lapatinib is a dual TKI that targets HER2/neu and EGFR mutations and is applied to advanced or metastatic breast cancer, vemurafenib and dabrafenib target BRAF mutations in melanoma, and crizotinib, ceritinib, and alectinib target anaplastic lymphoma kinase fusions in lung cancers (7, 81).
3. Biologics: recent studies have shown promising results with the administration of monoclonal antibodies and immune-modulating therapies, activating T-cell responses to target BM and LM in a non-cytotoxic manner. Bevacizumab is a monoclonal antibody that targets high levels of VEGF in several cancers to inhibit angiogenesis (109). Rituximab and trastuzumab are non-cytotoxic monoclonal antibodies that are administered intrathecally, targeting CD20 and HER2/neu, respectively (7); however, trastuzumab has been linked with increased incidence of brain metastasis. Checkpoint inhibitors such as ipilimumab and nivolumab are a new class of immunotherapies showing great promise in phase trials, targeting CTLA-4 and PD-L1 in metastasis of kidney, melanoma, and non-small cell lung cancer (6, 110). A more extensive coverage of the ongoing research on immunotherapies directed at BM is comprehensively discussed in the review by Farber et al. (111).

**Table 1:** List of selected chemotherapies, small molecules, and immunotherapies administered in the treatment of brain metastases (BM) and leptomeningeal metastases.

Drug	Mechanism of action	Mode of delivery	Clinical uses	Reference
**Chemotherapy**				

Cisplatin	Inhibitor of DNA replication	Intravenous infusion	Treatment of BM in patients with breast cancer, NSCLC, and melanoma	Franciosi et al. (82)

Cyclophosphamide	Inhibitor of DNA replication	Intravenous infusion; oral administration	Treatment of BM in patients with breast cancer	Rosner et al. (83)

Irinotecan	Inhibitor of DNA replication and transcription	Intravenous infusion	Treatment of BM in patients with small cell lung cancer	Sevinc et al. (84)

Methotrexate	S-phase specific cytotoxic chemotherapy	Intraventricular or intrathecal infusion	Treatment of patients with neoplastic meningitis	Grossman et al. (8)

Cytarabine (Ara-C)	Antimetabolite, blocks activity of DNA polymerase	Intraventricular or intrathecal infusion	Targeting leptomeningeal dissemination of patients with glioma, breast cancers, and NSCLC	Zhao et al. (85)
				Niwińska et al. (86)
	Disruption of PI3K/Akt/mTOR pathway			Nagpal et al. (87)

Thiotepa	Non-specific cell cycle inhibitor	Intraventricular or intrathecal infusion	Treatment of patients with neoplastic meningitis	Grossman et al. (8)

Capecitabine	Antimetabolite, blocks activity of DNA polymerase	Oral administration	Treatment of BM in patients with HER2-positive breast cancer	Petrelli et al. (88) Bachelot et al. (89)

**Small molecules**				

Gefitinib	Inhibitor of EGFR-associated tyrosine kinase	Oral administration	Targeting brain metastasis in patients with NSCLC	Ceresoli et al. (90)

Osimertinib	Inhibitor of EGFR-activating mutations and EGFR with T790M mutation	Oral administration	Targeting brain metastasis in NSCLC patients with EGFR T790M mutation	Koba et al. (91)

Erlotinib	Inhibitor of EGFR-associated tyrosine kinase	Oral administration	Targeting brain metastasis in patients with NSCLC	Sperduto et al. (92)

Lapatinib	Inhibitor of EGFR and HER2/neu	Oral administration	Targeting brain metastasis in patients with HER2+ breast cancer	Lin et al. (93)
				Lin et al. (94)

Vemurafenib	Inhibitor of BRAF, resulting in disruption of BRAF/MEK/ERK pathway	Oral administration	Targeting brain metastasis in patients with V600E *BRAF* mutation-positive melanoma	Dummer et al. (95)

Dabrafenib	Inhibitor of BRAF, resulting in disruption of BRAF/MEK/ERK pathway	Oral administration	Targeting brain metastasis in patients with V600E *BRAF* mutation-positive melanoma	Long et al. (96)

Crizotinib	Inhibitor of anaplastic lymphoma kinase (ALK) and c-ros oncogene 1 (ROS1)	Oral administration	Targeting brain metastasis in patients with EML4-ALK fusion NSCLC	Yoshida et al. (97)

Ceritinib	Inhibitor of ALK	Oral administration	Targeting brain metastasis in patients with ALK-positive NSCLC	Melosky et al. (98)

Alectinib	Inhibitor of ALK	Oral administration	Targeting brain metastasis in patients with ALK-positive NSCLC that are resistant to crizotinib	Gadgeel et al. (99)

**Biologics**				

Bevacizumab	Monoclonal antibody, blocks angiogenesis though inhibition of VEGF-A	Intravenous infusion	Targeting brain metastasis in patients with NSCLC	Besse et al. (100)

Rituximab	Monoclonal antibody targeting CD20	Intraventricular or intrathecal infusion	Targeting leptomeningeal dissemination of patients with lymphoma	Schulz et al. (101)

Trastuzumab	Monoclonal antibody targeting HER2/neu	Intravenous infusion or subcutaneous injection	Preventing brain metastasis development in HER2-overexpressing metastatic breast cancer	Park et al. (102)

Ipilimumab	Monoclonal antibody targeting CTLA-4	Intravenous infusion	Targeting brain metastasis in patients with melanoma	Margolin et al. (103)

Nivolumab	Monoclonal antibody targeting PD-1	Intravenous infusion	Targeting brain metastasis in patients with NSCLC	Dudnik et al. (104)

Pembrolizumab	Monoclonal antibody targeting PD-1	Intravenous infusion	Targeting brain metastasis in patients with melanoma or NSCLC	Goldberg et al. (105)

One of the major hurdles in developing novel therapies for brain tumors has been the paucity of overlapping actionable targets between treatment-naïve and recurrent tumors. Using a sleeping beauty transposon system in Ptch+/− mice with mutated *Trp53*, Wu et al. were able to generate GEMM of sonic hedgehog MB with increased tumor penetrance and reduced latency period (112). The introduction of humanized therapy protocols combining surgical resection and fractionated craniospinal irradiation led to generation of a mouse model that recapitulated both tumor initiation as well as disease progression, including rise of local and distal metastasis (113). The development of novel therapeutics is further complicated by the hurdles encountered throughout delivery of anticancer drugs to the brain. The BBB and BCSFB are substantial obstacles that need to be overcome to identify feasible cancer therapies. Both barriers differ in composition, permeability, and function. The BBB is a barrier formed by the endothelial cells of the brain capillaries, closely associated with pericytes, perivascular astrocytes, and microglia, and separates the circulating blood from the brain interstitial fluid (114). The BCSFB is composed of modified cuboidal epithelium of the choroid plexus, serving to secrete and separate CSF from circulating blood (114). Both barriers express transporters, multi-specific carriers, receptors, and enzymes that help to regulate diffusion and transport of polar molecules, essential nutrients, and wastes, and restrain the passage of anticancer compounds into the brain (114). Several factors within the composition of a drug also impede its ability to penetrate the BBB and BCSFB, including lipid solubility, molecular weight, polarity, and protein binding (7).

Various methods have been employed when designing drugs and delivery systems to increase drug efficacy in crossing the BBB and BCSF and targeting BM and LM. One method utilized to avoid these barriers is the use of transporters, where a drug that is not able to cross the BBB is coupled to a substance that can. This process can improve the peripheral pharmacokinetics, yet results in a hybrid compound that may not be recognized by the transporter or is destroyed as a foreign body (115). Another method is the formulation of a compound to be highly lipid soluble and with low molecular weight, increasing the likelihood of drug transport by transmembrane diffusion. Typical clinical therapeutic drugs are small, lipid soluble molecules. Unfortunately, once across the BBB the drug must then enter the surrounding aqueous interstitial fluid of the brain to be effective, resulting in drugs that are too lipid soluble being sequestered to the capillary bed and unable to reach areas beyond the BBB (116). The evidence of exosome-based communication in neural cells (117) opened up a possibility of potentially developing therapies that deliver short interfering RNA (siRNA) against specific targets to the brain. Despite a lack of clinical trial testing the efficacy of exosome-based therapies for cancer has been initiated, a study published by Alvarez-Erviti et al. (118) demonstrated a prominent reduction of both mRNA and protein levels of BACE1 within multiple brain cell lineages post siRNA delivery to the brain (118). Other delivery strategies include targeting peptides, regulatory proteins, and oligonucleotides (115).

## Conclusion

Brain metastases and LM are a common complication of cancer progression, associated with poor survival and limited treatment options. Elucidation of the molecular mechanisms underlying brain metastasis development, which includes the shifting microenvironment and interactions with the immune system the disseminated cell encounters, is supremely difficult to capture with an *in vitro* system. Consequently, experimental *in vivo* models are heavily relied on to serve as platforms to explore the nature of metastatic dissemination in a more comprehensive manner. Unique models have been generated with fish, mice, chicks, and companion animals (cats and dogs), all providing much needed knowledge to the field. However, there are several shortcomings associated with *in vivo* models, including the lack of feasible models that recapitulate the clinical progression of BM development in its entirety, and the obvious dissimilarities between the biological makeup of an animal and human. As such one must be aware of the benefits and caveats associated with available models to properly interpret results. Nonetheless, these animal models have provided significant knowledge of the characterization of metastatic disease progression in a live host and are a fundamental component to the identification, study, and testing of new cancer regimens.

## Author Contributions

Conceptualization of review, drafting of work, revision of manuscript, figure design, final approval of manuscript, and agreement for accountability for content of work: MS. Conceptualization of review, revision of manuscript, final approval of manuscript, and agreement for accountability for content of work: DB, CV, and SS.

## Conflict of Interest Statement

The authors declare that the research was conducted in the absence of any commercial or financial relationships that could be construed as a potential conflict of interest.
